# Malaria causes long-term effects on markers of iron status in children: a critical assessment of existing clinical and epidemiological tools

**DOI:** 10.1186/s12936-018-2609-6

**Published:** 2018-12-11

**Authors:** Filip C. Castberg, Edem W. Sarbah, Kwadwo A. Koram, Nicholas Opoku, Michael F. Ofori, Bjarne Styrishave, Lars Hviid, Jørgen A. L. Kurtzhals

**Affiliations:** 1 Centre for Medical Parasitology, Department of Immunology and Microbiology, Faculty of Health and Medical Sciences, University of Copenhagen, Copenhagen, Denmark; 2 Centre for Medical Parasitology, Department of Clinical Microbiology, Copenhagen University Hospital (Rigshospitalet), Copenhagen, Denmark; 3 Noguchi Memorial Institute for Medical Research, Accra, Ghana; 4 Hohoe Municipality Hospital, Hohoe, Ghana; 5 Present Address: School of Public Health, University of Health and Allied Sciences, Ho, Ghana; 6 Toxicology and Drug Metabolism Group, Department of Pharmacy, Faculty of Health and Medical Sciences, University of Copenhagen, Copenhagen, Denmark; 7 Centre for Medical Parasitology, Department of Infectious Diseases, Copenhagen University Hospital (Rigshospitalet), Copenhagen, Denmark

**Keywords:** Iron deficiency, Malaria, Hepcidin, FGF23, Ferritin

## Abstract

**Background:**

Most epidemiological studies on the interplay between iron deficiency and malaria risk classify individuals as iron-deficient or iron-replete based on inflammation-dependent iron markers and adjustment for inflammation by using C-reactive protein (CRP) or α-1-acid glycoprotein (AGP). The validity of this approach and the usefulness of fibroblast growth factor 23 (FGF23) as a proposed inflammation-independent iron marker were tested.

**Methods:**

Conventional iron markers and FGF23 were measured in children with acute falciparum malaria and after 1, 2, 4, and 6 weeks. Children, who were transfused or received iron supplementation in the follow-up period, were excluded, and iron stores were considered to be stable throughout. Ferritin levels 6 weeks after admission were used as a reference for admission iron status and compared with iron markers at different time points.

**Results:**

There were long-term perturbations in iron markers during convalescence from acute malaria. None of the tested iron parameters, including FGF23, were independent of inflammation. CRP and AGP normalized faster than ferritin after malaria episodes.

**Conclusion:**

Malaria may bias epidemiological studies based on inflammation-dependent iron markers. Better markers of iron status during and after inflammation are needed in order to test strategies for iron supplementation in populations at risk of malaria.

## Background

The geographical distributions of iron deficiency and malaria overlap. Iron deficiency has a negative impact on child development [1–4], but may also be associated with reduced susceptibility to infections because iron is essential for the growth of micro-organisms [5]. In line with this, an increasing number of epidemiological studies suggest that iron deficiency has a protective effect against malaria [6–10]. However, these studies have used conventional inflammation-dependant biomarkers, such as ferritin, as indicators of iron status, although they are known to be modified (usually increased) by malaria [11, 12]. The studies may therefore be biased due to iron status misclassification of study subjects.

Correspondingly, iron supplementation has been associated with increased risk of malaria, e.g., in the widely cited Pemba trial, which showed that routine iron supplementation resulted in increased morbidity and mortality among participating children [13]. Although some other studies have failed to confirm this finding [14], it remains a matter of dispute how to safely treat iron deficiency in areas where malaria is endemic.

Conventionally, serum ferritin has been used as the key iron marker as it reflects total body iron storage. However, ferritin is also an acute phase protein [15], so in an acute inflammatory situation, such as during malaria, serum ferritin levels may not accurately reflect body iron stores. In the present study, a time-series study of iron biomarkers was performed, as called for in the recent BRINDA study of anaemia in the context of inflammation [16]. Iron status was assumed to be stable during the study period for each study subject provided he or she did not receive a blood transfusion or iron supplementation. Thus, the intention was to use ferritin levels after normalization post infection as an indication of the true iron status during an acute malaria attack.

Using this reference point, the possibility that other iron markers during acute malaria might be correlated with ferritin after normalization was studied. The main focus was on hepcidin and fibroblast growth factor 23 (FGF23). Hepcidin is a peptide hormone that is regulated by iron stores, inflammation and erythropoietic demand and controls iron efflux from most cells in the body, in particular enterocytes and macrophages. Hepcidin has been suggested as the best marker to guide iron treatment as it has been proposed to be a key determinant of iron utilization [17, 18]. Yet, hepcidin was found to be a poor predictor of bone marrow iron deficiency and of iron incorporation in severely anaemic Malawian children [19]. FGF23, a bone-derived hormone regulating vitamin D and phosphate homeostasis, has been proposed as an inflammation-independent iron marker in a single epidemiological study [20]. In the present study, the aim was to investigate if FGF23 was independent of inflammation in acute malaria.

## Methods

### Ethical statement

The study was approved by the Noguchi Memorial Institute for Medical Research Institutional Review Board (NMIMR STC Number: STC Paper 5(1) 2013–2014) and by the Ethical Review Committee of the Ghana Health Service (file GHS-ERC 08/05/14). Parents/guardians of all study participants were informed in their local language, *Ewe*, of the goals, benefits and risks of taking part in the study, and written consent was obtained prior to enrolment.

### Study site and participants

The study was conducted in Hohoe, a town located about 220 km northeast of Accra, in an area of tropical, semi-deciduous, forest vegetation and used a convenience sample of children included in the MAVARECA study (Malaria Vaccine and Research Capacity Building in Ghana) [21]. Malaria transmission intensity in the area is high and has two seasonal peaks: a major one in April–July and a minor one in September–November [22]. Study participants were enrolled at Hohoe Municipal Hospital, June–August 2014 (pilot study) and June–August 2015.

Inclusion criteria were age 1–12 years, positive *Plasmodium falciparum* rapid diagnostic test (RDT), microscopic finding of peripheral parasitaemia > 2500 infected erythrocytes (IEs)/µL, fever (≥ 37.5 °C) within the first 24 h of admission or a history of fever in the preceding 24 h. Subjects were excluded if they had severe co-morbidity, including sickle-cell disease, or received a blood transfusion during admission due to severe anaemia (haemoglobin (Hb) < 5 g/dL) as the iron content of the transfused erythrocytes would otherwise distort the interpretation of the analysis of iron marker kinetics during follow up. During the follow-up period, patients were excluded if they presented with malaria, other severe disease or new spikes in inflammatory markers (C-reactive protein (CRP) > 5 mg/L or α-1-acid glycoprotein (AGP) > 1 g/L), or had taken iron supplementation (only excluded after such an event). The reason for excluding these patients was that these events would have affected day-42 results and thus the final endpoint.

Upon enrolment, a project nurse and physician completed a standardized questionnaire and performed a clinical examination. Severe malaria was defined according to WHO criteria [23]. Uncomplicated malaria cases were treated with a 3-day course of oral artemether–lumefantrine (AL), while severe malaria cases were treated with intravenous quinine for at least 24 h until oral AL was tolerated as follow-on therapy. In severe cases, ceftriaxone was given as empiric therapy for possible sepsis.

The patients were followed for 6 weeks and attended the research clinic 14 and 42 days post-admission (regular follow-up group). A sub-set of patients living near the hospital were also asked to come 7 and 28 days post-admission (frequent follow-up group). At follow-up visits, the patients or their parents were systematically interviewed about new symptoms and medicine use, including iron supplements, during the follow-up period. A missed follow-up appointment did not exclude the patient from future follow-up visits. Patients were encouraged to attend the research clinic at any time during the follow-up period if they developed any new symptoms.

For the purpose of this study, inflammation was defined as either CRP > 5 mg/L or AGP > 1 g/L, while iron deficiency was defined as ferritin concentrations < 15 µg/L on day 42.

### Laboratory methods

Venous blood (6 mL) was collected in lithium-heparin- and EDTA-coated tubes at each of the above time points. A WHO-approved RDT kit and Giemsa-stained blood smears were used to assess malaria infection status, and Hb (reference range 11–18 g/dL, coefficient of variation (CV) < 4%), mean corpuscular volume (MCV, 76–96 fL, CV < 5%), mean corpuscular Hb concentration (MCHC, 31–36 g/dL, CV < 6%), mean corpuscular Hb (MCH, 27–30 pg, CV < 5%), red cell distribution width (RDW-CV, < 14.6%, CV < 15%) were measured using a Sysmex XS 500i. Remaining plasma was separated by centrifugation and stored frozen at − 80 °C until further analysis. Plasma hepcidin concentrations (0.6–13.9 mmol/L, CV < 11%) were determined by mass spectrometry. Sample preparation was done in Eppendorf Lobind tubes (Sigma Aldrich, Søborg, Denmark) and all work was done under anaerobic conditions. Briefly, heparin plasma and internal standard [hep-25, Peptides International (Louisville, KY, USA)] was mixed with Macro-Prep^®^ CM Weak Cation Exchange (WCX) beads (Bio-Rad, Hercules, CA, USA) and ammonium acetate at pH 7.5. After washing, the analyte was eluted with 25 µL 2.5% Trifluoroacetic acid, 50% Acetonitrile LC–MS Chromasolv solution (both from Sigma-Aldrich) and the WCX beads were separated from supernatant by centrifugation at 500*g*. Peptide spectra were then generated on a Microflex matrix-enhanced laser desorption/ionization (MALDI) TOF–MS platform (Bruker Daltonics, Bremen, Germany). Levels of FGF23 (< 125 RU/mL, CV 2.4%) were measured in duplicates using a 2nd generation, C-terminal, two-site ELISA (Immutopics Inc, CA, USA). Biochemical parameters (reference ranges), including AGP (≤ 1 g/L), bilirubin (4–22 µmol/L), high sensitivity CRP (≤  5 mg/L), fe (5–30 µmol/L), ferritin (15–140 µg/L), haptoglobin (0.3–1.8 g/L), lactate dehydrogenase (LDH, 150–400 U/L), transferrin (18.7–50 µmol/L), and transferrin saturation (20–50%) were analysed on a Cobas 8000, (Roche, Rotkreuz, Switzerland, all CV < 7%). Levels of soluble transferrin receptor (sTfR, 0.76–1.76 mg/L, 3.6–4.3%) were measured (2014 samples only) in duplicates by BNII nephelometry (Siemens, Munich, Germany). Sickle cell Hb phenotype was determined by electrophoresis, while glucose-6-phosphate dehydrogenase (G6PD) deficiency was determined by methylene blue reduction test [24] (day 42 samples only).

Urine (> 12 mL) was collected at enrolment and tested for signs of microscopic haematuria and urinary tract infections using Siemens Multistix^®^10 SG. Microscopy analysis of all 2014 pilot urine samples yielded no positive results for *Schistosoma haematobium* eggs. In 2015, only samples with dipstick data suggesting haematuria were assessed by microscopy.

Stool samples (> 4 mL) were collected during admission. At least 2 mL were stored frozen (for later PCR detection of infection by *Cryptosporidium*, *Giardia lamblia* and *Entamoeba histolytica*), while at least 2 mL were mixed with formalin (10%) and stored at room temperature for later formalin-ether concentration (a.m. Ridley) and microscopy for cysts, helminth larvae and eggs.

### Statistical analysis

Data were double-entered in a Microsoft Access database. Mismatches were resolved by consultation of the original records. Statistical analyses were done in SAS v. 9.4 (SAS Institute, NC, USA). All continuous variables are presented as geometric means ± 2 standard deviations (SD), unless otherwise specified. Longitudinal data were analysed using log-transformed variables in a mixed effects model. P values < 0.05 were considered statistically significant. As some CRP values were 0, i.e., below the lower detection level of 1, “1” was added to the CRP value before log transformation. Pearson’s correlation coefficient (R) was used to determine correlation between log-transformed iron markers.

Only data collected ± 2 days from the scheduled follow-up date were included, except for day 42, where delays in data collection were tolerated (six samples were collected after day 44, the latest on day 51).

## Results

**Fig. 1:**
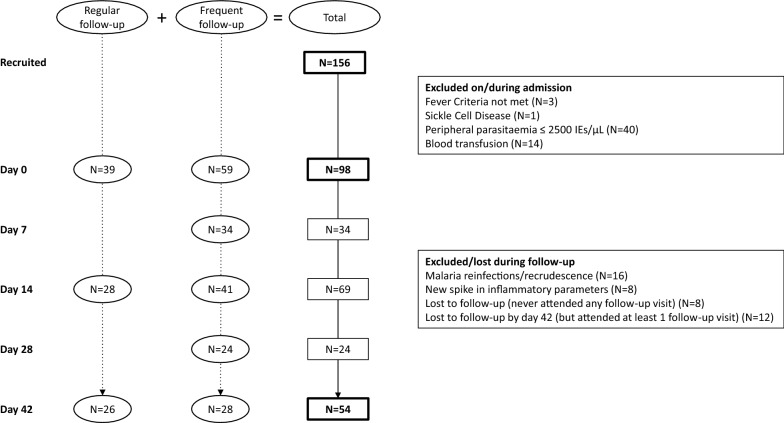
Study flow chart. Study profile of patients who were recruited, enrolled in, and completed the study

A total of 156 patients with a positive RDT were recruited to participate in the study. Three patients did not meet the fever criteria, one was excluded due to sickle cell disease, 40 had ≤ 2500 IEs/µL on presentation, and 14 patients received a blood transfusion during admission on clinical indication or because their Hb levels decreased to < 5 g/dL. The analysis is restricted to the 98 remaining patients (Fig. 1). A summary of the study population characteristics can be found in Table 1.

**Table 1:** Study population characteristics

Description	N	Value
Child characteristics		
Age (geometric mean, range)	97	3.8 years (1; 12)
Age < 60 months	97	63
Female	98	44
Nutritional status		
Height (mean ± 2SD)	71	98 cm (69.2; 128.6)
Weight (geometric mean ± 2SD)	98	14.4 kg (7.7; 26.6)
Stunted (children < 60 months)^a^	45	13 (29%)
Underweight (children < 60 months)^a^	63	11 (17%)
Low BMI-for-age (children > 60 months)^b^	26	8 (31%)
Admission		
Hospital admission (yes/no)	98	60/38
Admission length (median, IQR)	60	3 days (2; 4)
Clinical malaria (uncomplicated/severe)	98	68/30
Clinical co-infection on presentation (yes/no)^c^	98	22/76
Laboratory parameters		
Malaria parasitaemia on presentation (geometric mean ± 2SD)	98	40,326 IEs/µL (3465; 469,356)
Haemoglobin on presentation (geometric mean ± 2SD)	98	9.5 g/dL (6.7; 13.5)
Anaemia level on presentation (no anaemia/mild/moderate)^d^	98	24/60/14
Iron deficiency on day 42 (ferritin < 15 µg/L)	52	4
Glucose-6-phosphate dehydrogenase deficiency (normal enzyme activity/partial defect/full defect)^e^	54	39/4/11
Sickle cell genotype (AA/AC/AF/AS)	98	85/8/2/3
Raised C-reactive protein on day 0 (> 5 mg/L)	94	93
Raised α-1-acid glycoprotein on day 0 (> 1 g/L)	92	90
Positive stool microscopy for anaemia-causing pathogenic organisms^f^	74	4
Antimalarial treatment prior to hospital attendance	98	19
Antimalarial treatment prescribed (quinine i.v. + artemether–lumefantrine follow-on/artemether–lumefantrine only)	98	44/54

N = Number of children; Value = Number of children unless otherwise specified

^a^Stunting and underweight are defined as height-for-age and weight-for-age, respectively, < − 2 standard deviations of the WHO Growth Reference [46]

^b^Low BMI-for-age is defined as BMI-for-age < − 2 standard deviations of the WHO Growth Reference [46]

^c^Dermatitis (N = 2), gastroenteritis (N = 10), lower respiratory tract infections (N = 3), suspected sepsis (N = 2), upper respiratory tract infections (N = 2), urinary tract infections (N = 3)

^d^No anaemia (Hb ≥ 11 g/dL), mild anaemia (Hb 8–11 g/dL), moderate anaemia (Hb 5–8 g/dL)

^e^Tested on day 42

^h^Hookworm, *Hymenopelis nana, Schistosoma mansoni or Strongyloides stercoralis*. No children were excluded from the study based on stool findings

Twenty-four patients were excluded during follow-up: 16 had re-infection or recrudescence, while 8 developed new spikes in inflammatory markers. Eight patients never attended a follow-up visit, and 12 other patients were lost to follow-up by day 42. Fifty-four patients, who had normal inflammatory marker levels on day 42 were retained in the study (Fig. 1). No statistical differences in age, gender and iron or inflammatory parameters on day 0 were detected between the 54 patients who completed the study and the 44 who did not. Similarly, no significant differences in gender and iron or inflammatory parameters on day 0, 14 and 42 were noted between the regular follow-up group (N = 39) and the frequent follow-up group (N = 59). Patients scheduled for frequent follow-up were 1 year older (geometric mean age 4.2 vs 3.3, P = 0.03), but had similar gender distribution compared with the children in the regular follow-up group. Twenty-one samples were excluded as they were taken outside the predefined follow-up window (± 2 days of scheduled visits).

**Fig. 2:**
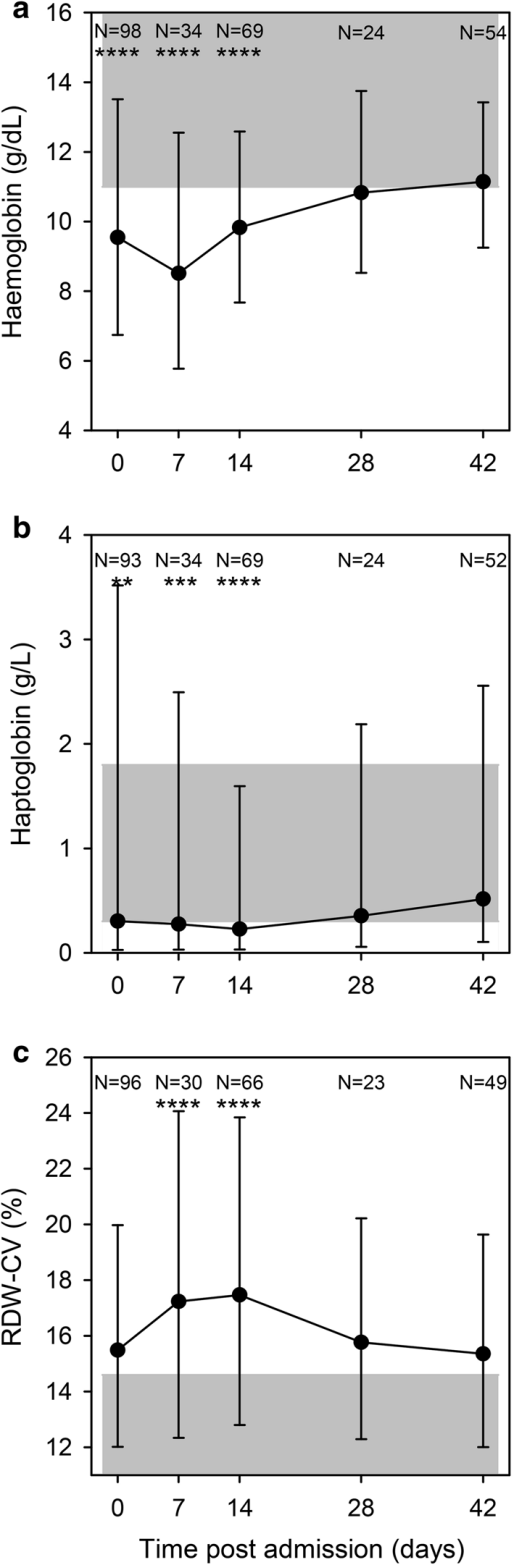
Haematology. Haemoglobin (**a**), haptoglobin (**b**), and red cell distribution width-coefficient of variation (RDW-CV) (**c**) on days 0, 7, 14, 28, and 42 post-admission. Geometric means (filled circle) and standard deviations (bars) are shown. Number of samples (N) and statistically significant differences (*P < 0.05, **P < 0.01, ***P < 0.001, ****P < 0.0001) relative to day 42 are indicated along the top of each panel. Normal reference area is indicated by grey shading

**Fig. 3:**
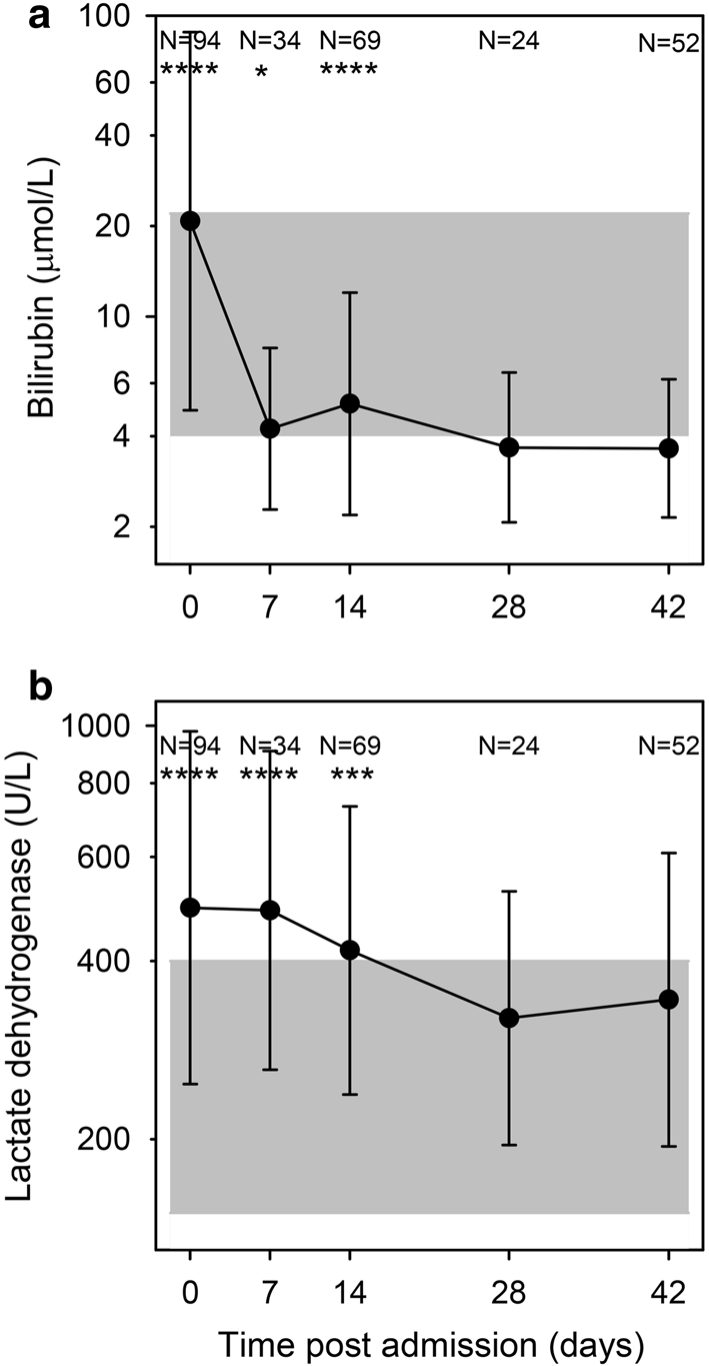
Bilirubin and lactate dehydrogenase. Plasma levels of bilirubin (**a**) and lactate dehydrogenase (**b**) on days 0, 7, 14, 28, and 42 post-admission. Geometric means (filled circle) and standard deviations (bars) are shown. Number of samples (N) and statistically significant differences (*P < 0.05, **P < 0.01, ***P < 0.001, ****P < 0.0001) relative to day 42 are indicated along the top of each panel. Normal reference area is indicated by grey shading

On admission, 76% of the included patients were mildly (Hb 8–11 g/dL) or moderately (Hb 5–8 g/dL) anaemic. (Patients with severe anaemia (Hb < 5 g/dL) were excluded). Hb levels decreased until day 7 post-admission despite treatment, but had returned to normal levels by day 28 (Fig. 2a). Indicators of haemolysis (low haptoglobin levels, Fig. 2b) and high levels of bilirubin (Fig. 3a) and lactate dehydrogenase (LDH) (Fig. 3b) followed this pattern. Erythropoiesis appeared to be suppressed (low RDW) at admission, and was followed by signs of increased erythropoiesis on day 7 (Fig. 2c), in agreement with an earlier study [25].

**Fig. 4:**
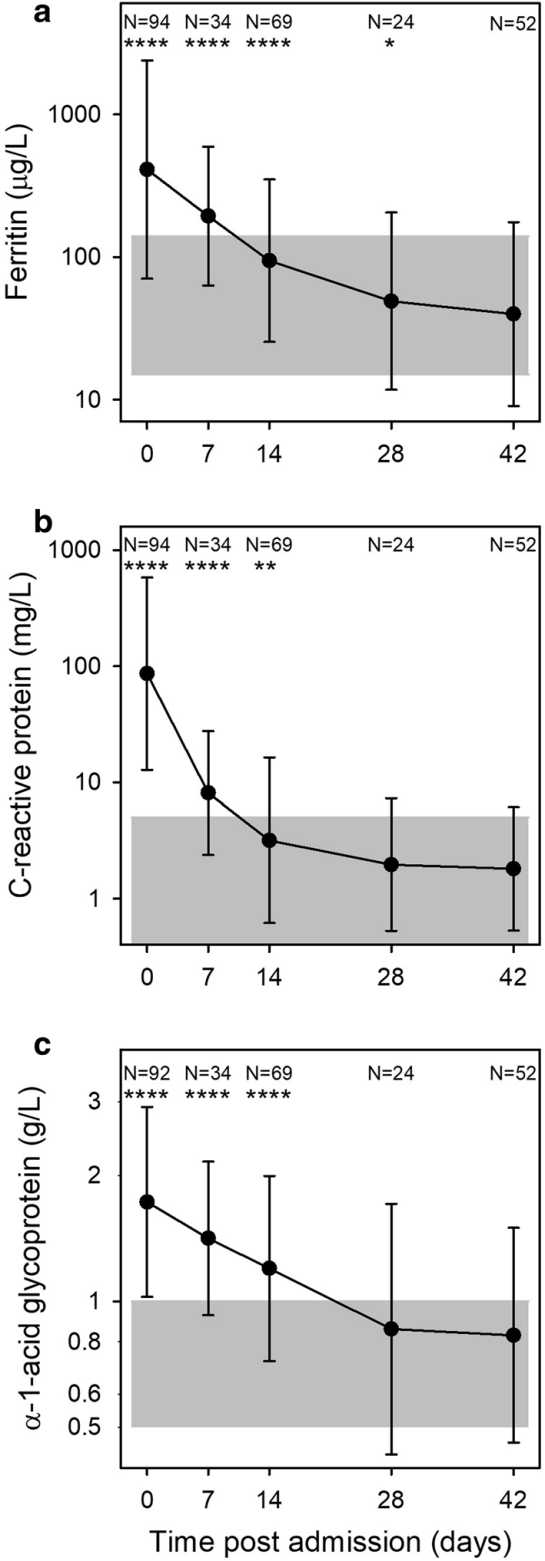
Ferritin and inflammation markers. Plasma levels of ferritin (**a**), C-reactive protein, (**b**), and α-1-acid-glycoprotein (**c**) on days 0, 7, 14, 28, and 42 post-admission. Geometric means (filled circle) and standard deviations (bars) are shown. Number of samples (N) and statistically significant differences (*P < 0.05, **P < 0.01, ***P < 0.001, ****P < 0.0001) relative to day 42 are indicated along the top of each panel. Normal reference area is indicated by grey shading

Ferritin levels were high on day 0 and gradually decreased during follow-up, including a statistically significant drop between day 28 and day 42 (Fig. 4a). Ferritin levels on day 42 had been selected as an indicator of the iron status throughout the study period, and the kinetics curve (Fig. 2a) supported the assumption that decreases beyond that day would be minimal.

Four of the children (8%) were iron-deficient (ferritin concentrations below < 15 µg/L on day 42). The acute phase proteins CRP (Fig. 4b) and AGP (Fig. 4c) are commonly used to compensate for the effect of inflammation when ferritin levels are used to detect iron deficiency [16, 26, 27]. Similar to ferritin, they were raised on day 0 and gradually fell, but they normalized faster than ferritin with no detectable difference between day 28 and day 42 levels.

**Fig. 5:**
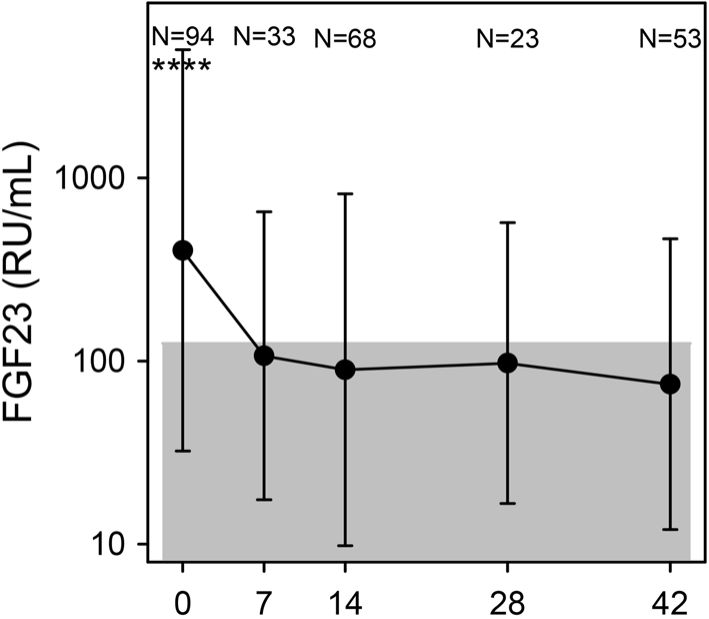
Fibroblast growth factor 23 (FGF23). Plasma levels of FGF23 on days 0, 7, 14, 28s and 42 post-admission on days 0, 7, 14, 28, and 42 post-admission. Geometric means (filled circle) and standard deviations (bars) are shown. Number of samples (N) and statistically significant differences (*P < 0.05, **P < 0.01, ***P < 0.001, ****P < 0.0001) relative to day 42 are indicated along the top of each panel. Normal reference area is indicated by grey shading

FGF23 levels were significantly elevated on day 0, and thus not independent of inflammation as previously claimed [20]. Day 0 levels of FGF23 showed a dichotomized distribution and were poorly correlated with CRP and AGP levels [R = 0.05 (P = 0.03), R = 0.03 (P = 0.08), respectively]. Levels had normalized by day 7 and remained stable throughout the remaining observation period (Fig. 5). Yet, even after it had normalized, FGF23 levels on days 14, 28 and 42 remained poorly correlated with ferritin levels on day 42 [R = − 0.58 (P < 0.0001), R = − 0.56 (P = 0.02), and R = − 0.51 (P = 0.0001), respectively].

**Fig. 6:**
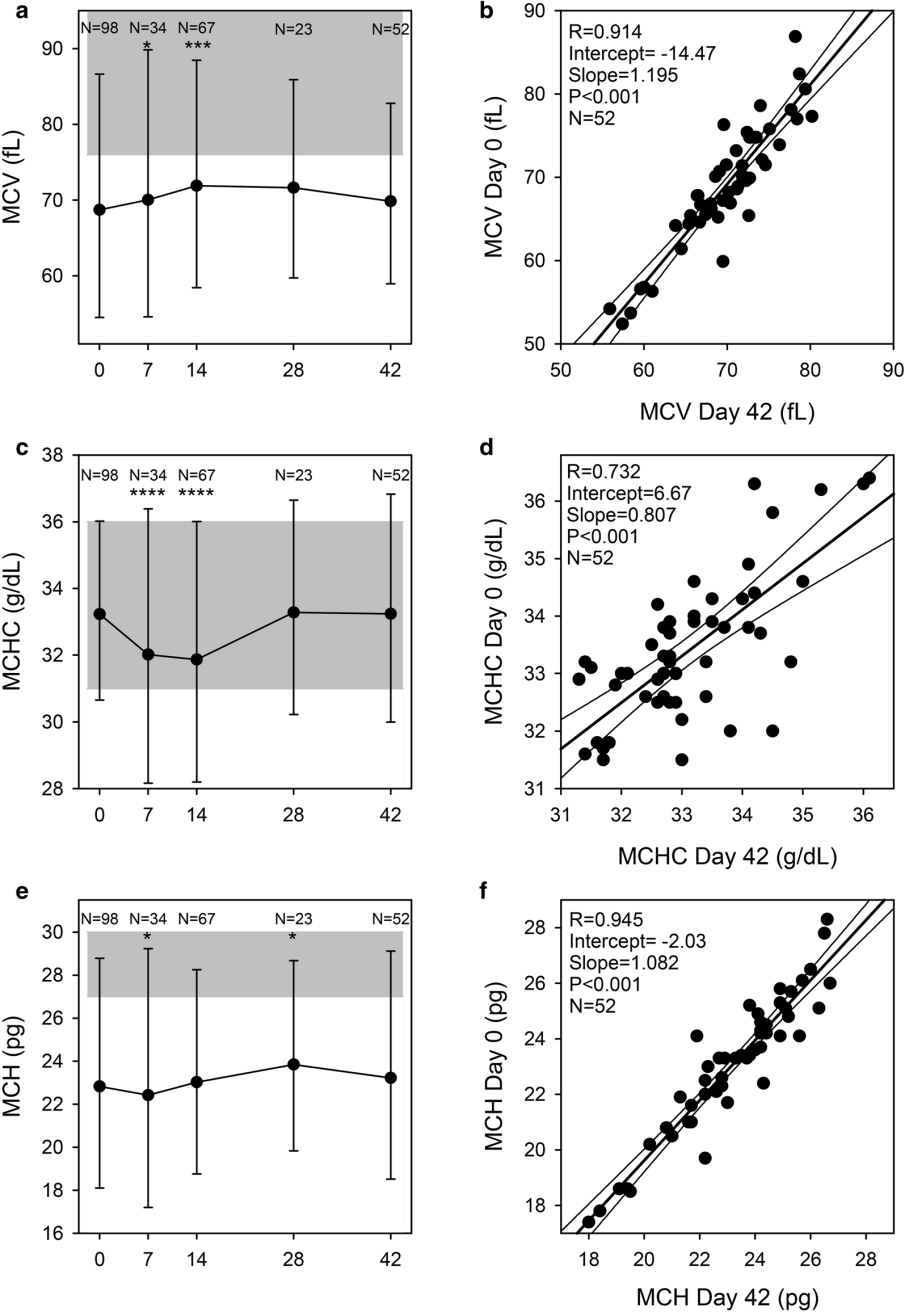
Red cell indices. Mean corpuscular volume (MCV) (**a**), mean corpuscular haemoglobin concentration (MCHC) (**c**), and mean corpuscular haemoglobin (MCH) (**e**) on days 0, 7, 14, 28 and 42 post-admission. Geometric means (filled circle) and standard deviations (bars) are shown. Number of samples (N) and statistically significant differences (*P < 0.05, **P < 0.01, ***P < 0.001, ****P < 0.0001) relative to day 42 are indicated along the top of each panel. Normal reference area is indicated by grey shading. Linear correlation of admission (day 0) and steady-state (day 42) data (**b**, **d**, **f**). Correlation between MCV, MCHC and MCH levels at admission (day 0) and at steady-state (day 42) (**b**, **d**, **f**). Individual data points (filled circle), and the associated linear regression line (with 95% confidence interval) are shown. The linear correlation coefficient (R^2^), its statistical significance, and the number of data points are indicated in the panel margin

Classic markers used in anaemia work-up, such as MCV (Fig. 6a), MCHC (Fig. 6c) and MCH (Fig. 6e), were all stable throughout the observation period and the regression analysis between day 0 and day 42 values were close to the identity line (Fig. 6b, d, f), indicating that measurements on day 0 were predictive of measurements on day 42 on study subject level. One MCHC measurement deviated far from all others without any other noticeable characteristics of this patient. This value was considered a measurement error and was omitted in the data analysis.

Malaria causes haemolytic anaemia that does not affect MCV, MCHC or MCH. Nevertheless, these markers all correlated poorly with ferritin levels on day 42 [MCV: R = 0.26 (P = 0.07); MCH: R = 0.30 (P = 0.03), MCHC: R = 0.10 (P = 0.49)]. Furthermore, the slightly increased MCV values day 7 to day 28 (Fig. 6a) and the concomitant decrease in MCHC levels (Fig. 6c) likely reflect increased erythropoiesis during this period as indicated by the raised RDW on days 7 and 14, (Fig. 2c).

**Fig. 7:**
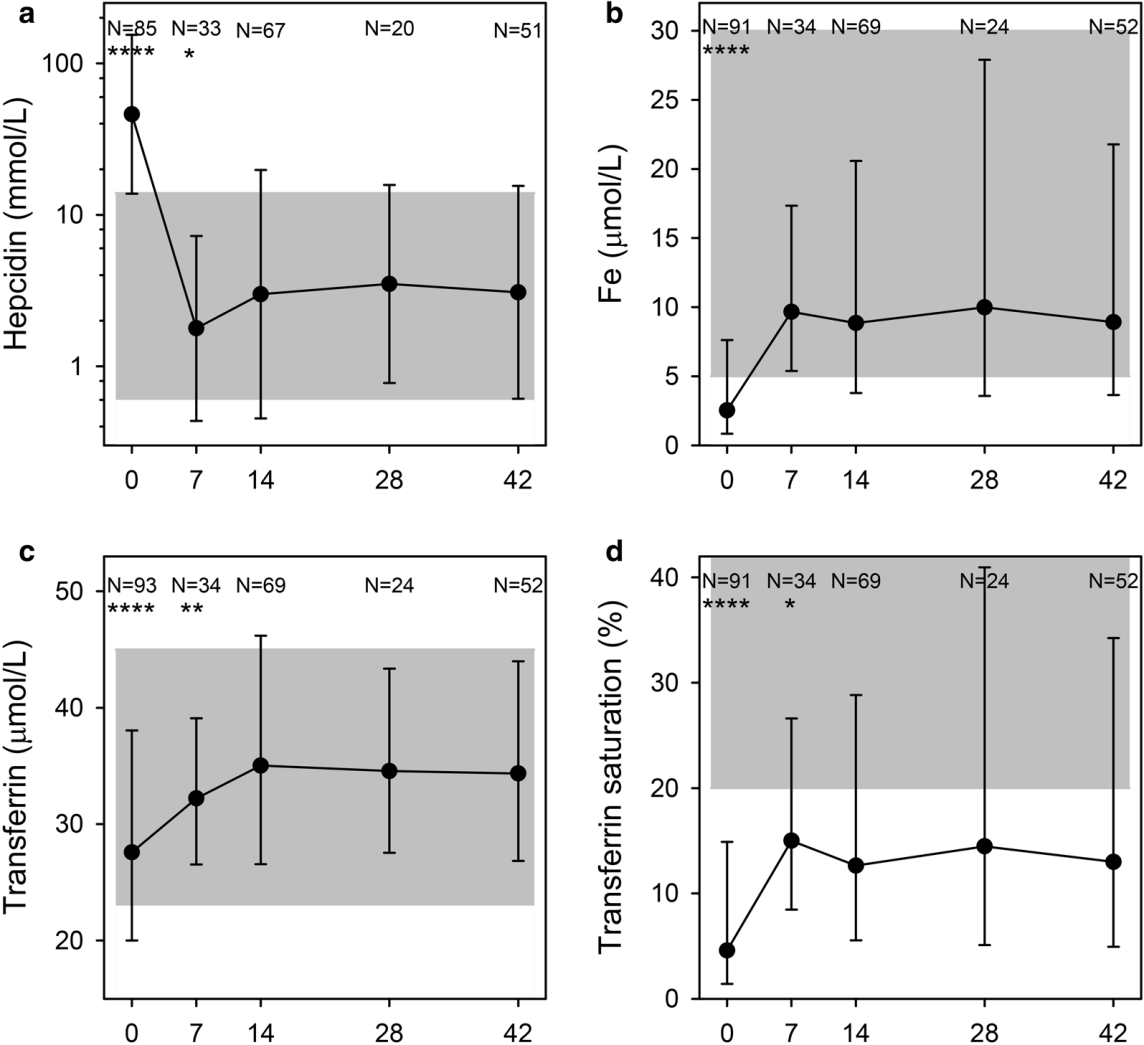
Additional iron markers. Plasma levels of hepcidin (**a**), iron (Fe) (**b**), transferrin (**c**), and transferrin saturation (**d**) on days 0, 7, 14, 28, and 42 post-admission. Geometric means (filled circle) and standard deviations (bars) are shown. Number of samples (N) and statistically significant differences (*P < 0.05, **P < 0.01, ***P < 0.001, ****P < 0.0001) relative to day 42 are indicated along the top of each panel. Normal reference area is indicated by grey shading

**Fig. 8:**
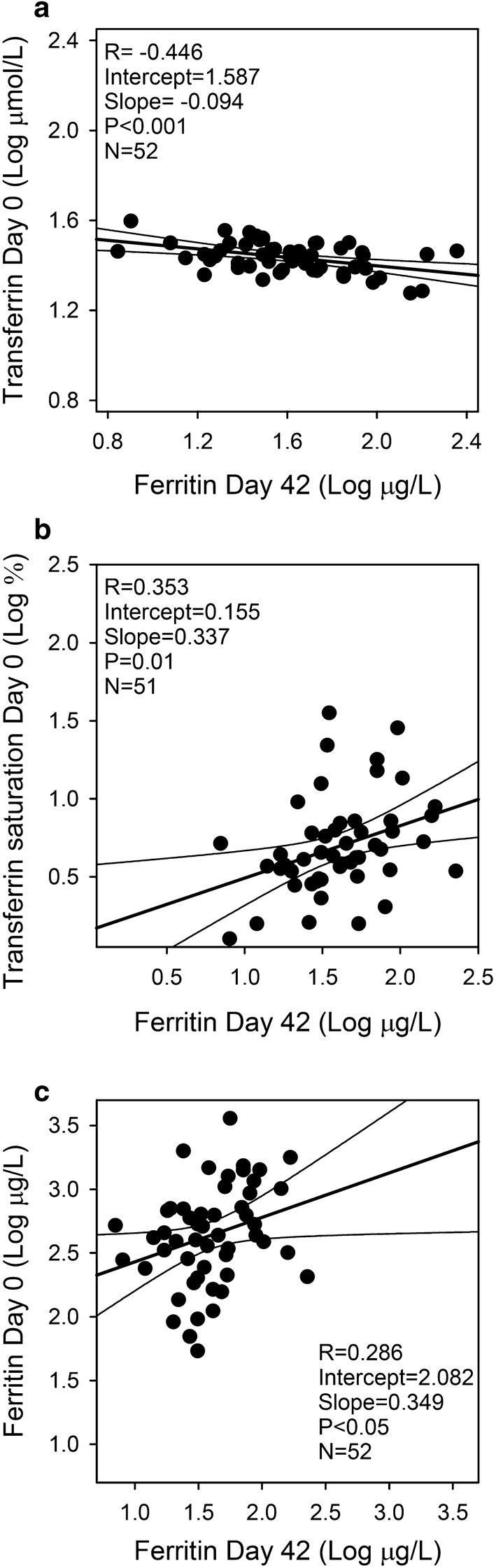
The three iron markers with best day 0 correlations to ferritin levels on day 42. Linear regression plots of log-transformed values of transferrin (**a**), transferrin saturation (**b**) and ferritin (**c**) on admission (day 0) vs log-transformed ferritin levels at steady-state (day 42). Individual data points (filled circle), and the associated linear regression line (with 95% confidence interval) are shown. The linear correlation coefficient (R^2^), its statistical significance, and the number of data points are indicated in the panel margin

During the acute malaria attack (day 0), hepcidin levels were raised (Fig. 7a), which was reflected in low levels of Fe, transferrin and transferrin saturation (Fig. 7b–d). Levels of hepcidin, Fe, transferrin, as well as transferrin saturation had normalized by day 14. Of all iron parameters, day 0 values of transferrin, transferrin saturation and ferritin had the best—yet weak—correlation with ferritin levels on day 42 [R = − 0.45 (P = 0.001), R = 0.35 (P = 0.01), and R = 0.29 (P = 0.04), respectively] (Fig. 8a–c).

In the 2014 pilot study, sTfR, an indicator of erythropoietic intensity, was also measured on days 0, 14 and 42. Day 0-levels of sTfR levels were below the day 42 levels (P = 0.0004), whereas day 14 levels were above day 42 levels (P = 0.03), consistent with post-malarial erythropoiesis. sTfR/log ferritin ratios were not associated with ferritin levels on day 42, why this parameter was omitted in the 2015 study.

On admission, patients with severe malaria had lower Hb (8.5 vs 10.1 g/dL, P < 0.0001), lower haptoglobin [0.16 vs 0.36 g/L, (P = 0.0001)], higher LDH [639 vs 445 U/L (P < 0.0001)], and higher bilirubin [26 vs 19 µmol/L (P = 0.005)], compared to patients with uncomplicated malaria, consistent with more pronounced haemolysis in the severe malaria group. Also, admission levels of ferritin and FGF23 were higher in patients with severe malaria [643 vs 365 µg/L (P = 0.002) and 647 vs 333 RU/mL (P = 0.02), respectively], likely due to inflammation. By day 7, these differences were no longer detectable, except differences in haemoglobin levels that did not resolve until day 28.

## Discussion

In this observational, longitudinal study, conventional iron markers as well as FGF23 were measured in children on admission with acute falciparum malaria and after 1, 2, 4 and 6 weeks. The most striking finding was the duration of iron marker perturbations following episodes of falciparum malaria. Ferritin was elevated for more than 4 weeks after the infection in children without evidence of recrudescence or re-infection (Fig. 4a). This finding challenges the interpretation of previous epidemiological studies of iron deficiency in malaria-exposed populations, which have used various approaches to control for inflammation. Some studies have employed an upward adjustment of the cut-off for ferritin in case of raised CRP to levels > 8.2–10 mg/L [7, 8], while others have used arithmetic correction factors based on CRP and AGP levels [10, 28, 29]. In the recent BRINDA study, it is advocated to use internal linear regression to correct ferritin concentrations based on CRP and AGP levels [27]. The fact that both CRP and AGP (Fig. 4b, c) normalized at least 2 weeks earlier than ferritin (Fig. 4a) suggests that this practice may not be sufficient to fully compensate for the effect of malaria on ferritin.

The only biomarkers of relevance to iron deficiency that were unaffected by the malaria-induced inflammation were MCV, MCHC and MCH (Fig. 6a, c, e). These remained stable throughout the study period with the exception of temporary changes associated with erythropoiesis. This supports the assumption that the iron status of the children did not change markedly over the 6-week follow-up period and that the ferritin levels on day 42 are likely to reflect the true iron status on day 0. However, MCV, MCHC and MCH all correlated poorly with ferritin and are thus unlikely to be useful as markers of iron status in the absence of overt iron deficiency anaemia.

It could be argued that a further drop in ferritin may occur after day 42. The time to normalization of ferritin after a range of clinical conditions has varied from 1 to 7 weeks, although this has not specifically been studied in malaria [30–33]. Forty-two days’ follow-up time was selected, which is the standard follow-up time used in studies of anti-malarial drug resistance. Although the curve had clearly flattened at day 42, the possibility of a longer lasting perturbation of ferritin following malaria cannot be ruled out, which would further question the use of ferritin as an indicator of iron deficiency in areas where malaria is endemic.

FGF23 is a bone-derived hormone involved in calcium-phosphate homeostasis regulated by active vitamin D and phosphate, and more recently also noted to be stimulated by iron deficiency [34]. In The Gambia, a population study found that FGF23 was associated with iron status in children independently of inflammation (defined as elevated CRP) [20]. However, in the present study, FGF23 was markedly elevated in acute malaria (Fig. 5). This finding is in line with recent studies in humans [35] and in experimental malaria [36]. Moreover, even though FGF23 levels normalized already on day 7, i.e., much earlier than ferritin levels, FGF23 levels correlated poorly with day-42 plasma ferritin. Hence, it does not seem promising to further explore the possibility of using FGF23 as an inflammation-independent indicator of iron status.

Among the other iron markers tested in this study (day 0 values), transferrin showed the best correlation with ferritin on day 42 [R = − 0.45 (P = 0.001)]. However, transferrin was also markedly affected by inflammation, and the correlation between transferrin day 42 levels with plasma ferritin levels on day 42 was modest. In the pilot study, sTfR levels were also evaluated. This biomarker is less affected by inflammation than ferritin but increases in haemolytic anaemia [37, 38]. Levels of sTfR are also directly associated with parasitaemia, and its usefulness in acute malaria studies has therefore been questioned [39]. The present study data supports this concern. The wide variation among values obtained with different sTfR test kits are also of concern [17]. The ratio of soluble transferrin receptor to log ferritin concentrations (sTfR/log ferritin index) has been suggested as a more precise iron marker, applying the reciprocal relationship between ferritin and sTfR [40], but the index suffers from the disadvantages of its parameters, i.e., their dependence of inflammation (ferritin) and erythropoiesis (sTfR).

The possibility that artemisinin-associated haemolysis might have enhanced perturbations of markers of erythropoiesis in the follow-up period was also considered. This condition is associated with hyperparasitaemia at the initiation of treatment [41]. In the present study, only 5 of 98 patients had over 250,000 malaria parasites/µL on admission, and they all had higher concentrations of LDH on admission than at any time in the follow-up period, while haptoglobin remained below the detection threshold until day 42. Hence, artemisinin-associated haemolysis is unlikely to have affected the overall results.

The lack of an inflammation-independent marker of iron deficiency may restrict the possibility to develop innovative strategies for iron supplementation in malaria-endemic areas. Experimental studies suggest that the increased susceptibility to malaria in the course of iron supplementation is temporary [42]. In order to shorten a possible window of vulnerability, rapid reconstitution of iron stores with the use of intravenous ferric carboxymaltose has previously been suggested [43]. This approach is supported by recent studies indicating that iron deficiency increases malaria mortality in mice and that intravenous iron given during acute malaria improves survival [36]. However, intravenous iron can cause iron overload, which is a safety hazard. Consequently, safe administration of intravenous iron requires targeting of patients with iron deficiency, and this would require an inflammation-independent marker for it to be used in children at risk of malaria.

The observed temporal changes in hepcidin levels are in line with previous studies [44]. During the acute phase of malaria, hepcidin was raised, indicating that inflammatory stimuli outweighed signals from the haemolytic anaemia of malaria. The drop in hepcidin levels day 7 (Fig. 7a) coincided with signs of increased erythropoiesis (Fig. 2c) and increased levels of plasma iron (Fig. 7b), and hepcidin remained low throughout the follow-up period. The low levels of hepcidin on day 7 indicate that oral iron supplementation would be effective shortly after recovery from malaria [45] and supports using hepcidin levels to guide iron therapy [17], although it cannot be used as a stand-alone marker of iron levels.

## Conclusion

Better biomarkers for iron stores in acute malaria are still needed to improve the understanding of the interplay between iron status and malaria and to develop safe strategies for iron supplementation in areas where malaria is endemic.

## Abbreviations

AGP: α-1-acid glycoprotein
AL: artemether–lumefantrine
CRP: C-reactive protein
FGF23: fibroblast growth factor 23
G6PD: glucose-6-phosphate dehydrogenase
Hb: haemoglobin
IEs: infected erythrocytes
MCH: mean corpuscular haemoglobin
MCHC: mean corpuscular haemoglobin concentration
MCV: mean corpuscular volume
RDT: rapid diagnostic test
RDW-CV: red cell distribution width (coefficient of variation)
sTfR: soluble transferrin receptor
