# Correction to: Malaria causes long-term effects on markers of iron status in children: a critical assessment of existing clinical and epidemiological tools

**DOI:** 10.1186/s12936-022-04117-6

**Published:** 2022-04-07

**Authors:** Filip C. Castberg, Edem W. Sarbah, Kwadwo A. Koram, Nicholas Opoku, Michael F. Ofori, Bjarne Styrishave, Lars Hviid, Jørgen A. L. Kurtzhals

**Affiliations:** 1 Department of Immunology and Microbiology, Faculty of Health and Medical Sciences, Centre for Medical Parasitology, University of Copenhagen, Copenhagen, Denmark; 2 Department of Clinical Microbiology, Centre for Medical Parasitology, Copenhagen University Hospital (Rigshospitalet), Copenhagen, Denmark; 3 Noguchi Memorial Institute for Medical Research, Accra, Ghana; 4 Hohoe Municipality Hospital, Hohoe, Ghana; 5 Present Address: School of Public Health, University of Health and Allied Sciences, Ho, Ghana; 6 Toxicology and Drug Metabolism Group, Department of Pharmacy, Faculty of Health and Medical Sciences, University of Copenhagen, Copenhagen, Denmark; 7 Centre for Medical Parasitology, Department of Infectious Diseases, Copenhagen University Hospital (Rigshospitalet), Copenhagen, Denmark

## Correction to: Malar J (2018) 17:464 https://doi.org/10.1186/s12936-018-2609-6

Following publication of the original article [1], the authors flagged that the article had published with an incorrect version of Fig. 7, and that in the ‘Laboratory methods’ subsection of the Methods the unit for hepcidin (‘**n**mol/L’) had been miswritten as ‘**m**mol/L’.

These errors have now been corrected in the original article and the corrected figure may be found in this correction. Regarding the figure, the authors would like to highlight to the reader that the N values for hepcidin in Fig. 7 have been corrected and that at hepcidin Day 7, the p-value symbol has been corrected from "*" to "**".

**Fig. 7:**
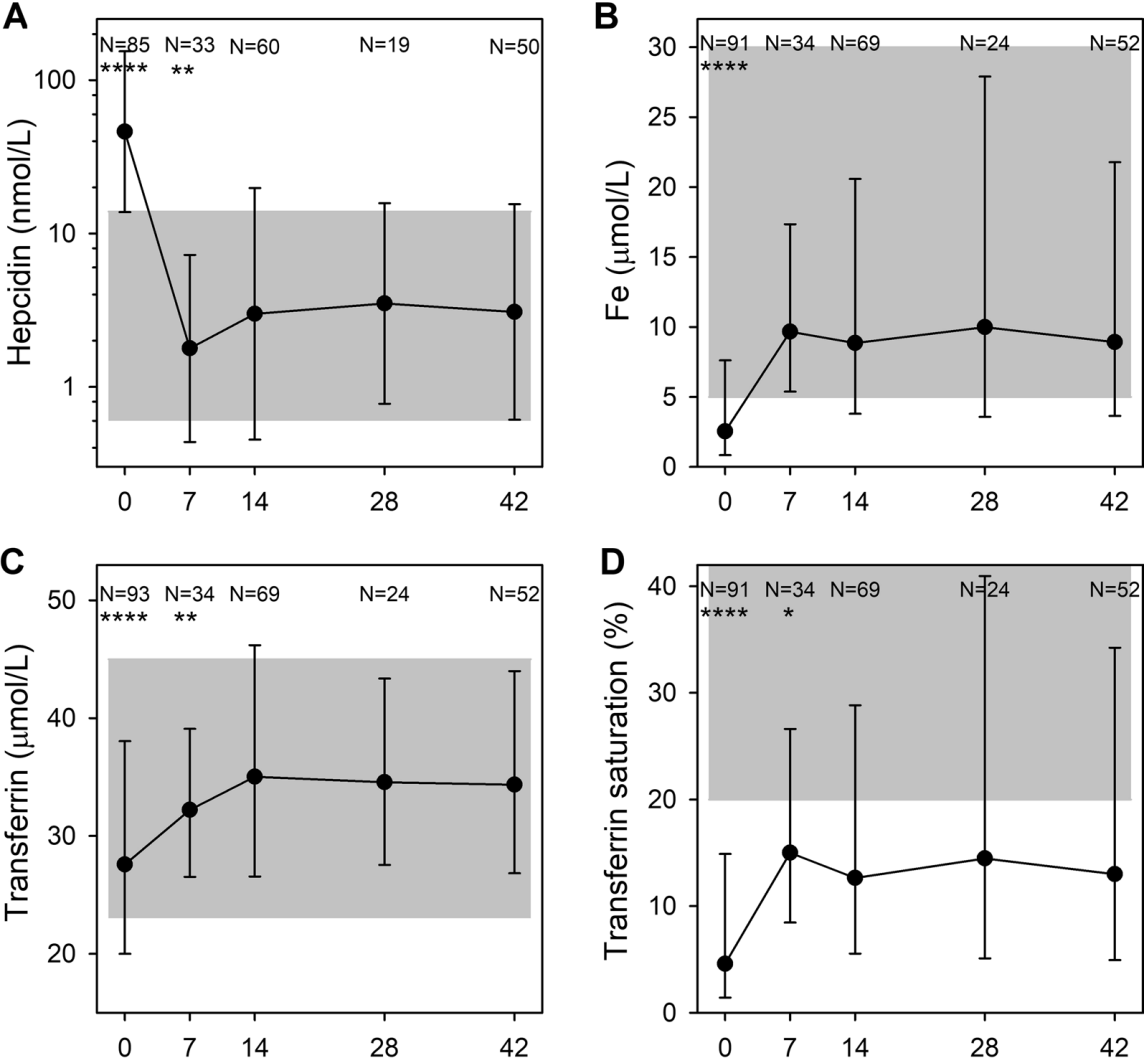
Additional iron markers. Plasma levels of hepcidin (**a**), iron (Fe) (**b**), transferrin (**c**), and transferrin saturation (**d**) on days 0, 7, 14, 28, and 42 post-admission. Geometric means (filled circle) and standard deviations (bars) are shown. Number of samples (N) and statistically significant differences (*P < 0.05, **P < 0.01, ***P < 0.001, ****P < 0.0001) relative to day 42 are indicated along the top of each panel. Normal reference area is indicated by grey shading
